# Tension between the need for certainty and numerous uncertainties—A focus group study on various perspectives on a potential genomic newborn screening program in Germany

**DOI:** 10.1002/jgc4.70004

**Published:** 2025-05-16

**Authors:** Elena Sophia Doll, Julia Mahal, Karla Alex, Seraina Petra Lerch, Stefan Kölker, Christian P. Schaaf, Eva C. Winkler, Beate Ditzen

**Affiliations:** ^1^ Ruprecht‐Karls University Heidelberg Heidelberg Germany; ^2^ Institute of Medical Psychology Heidelberg University Hospital Heidelberg Germany; ^3^ National Center for Tumor Diseases (NCT), NCT Heidelberg, a Partnership between DKFZ and Heidelberg University Hospital, Section Translational Medical Ethics, Department of Medical Oncology, Medical Faculty Heidelberg Heidelberg Germany; ^4^ Department of Medical Psychology University Medicine Greifswald Greifswald Germany; ^5^ German Center for Child and Adolescent Health (DZKJ), Partner Site Greifswald/Rostock Greifswald Germany; ^6^ Heidelberg University, Medical Faculty, Center for Child and Adolescent Medicine, Division of Child Neurology and Metabolic Medicine Heidelberg Germany; ^7^ Institute of Human Genetics, Heidelberg University, Medical Faculty Heidelberg Heidelberg Germany; ^8^ German Centre for Mental Health, Partner Site Heidelberg‐Mannheim‐Ulm Mannheim Germany

**Keywords:** ethics, genomic newborn screening, parents, population screening, predictive genetic testing, psychosocial

## Abstract

The advancement of genome sequencing technology and its potential application in newborn screening is being discussed in various countries. Genomic newborn screening (gNBS) can provide parents with information about their child's genetic susceptibility for known disorders. However, it also presents ethical and psychosocial challenges. This study was carried out with a view toward the possible introduction of gNBS in Germany. Due to the existing challenges, it is crucial to understand different perspectives of relevant groups in Germany before implementing gNBS. Four online focus groups were conducted with parents, patient representatives, and healthcare professionals to explore perceived opportunities and challenges, as well as needs regarding a potential gNBS program. Discussions with altogether 24 participants were semi‐structured using a pre‐defined interview guide. Sessions were audio‐visually recorded and transcripts were analyzed using a structuring qualitative content analysis combining both deductive and inductive methods. Participants expressed positive views about gNBS but also reservations about a gNBS program and posed requirements for operating conditions. One salient theme that emerged was hope for certainty through gNBS in the face of numerous uncertainties. The study complements the perspective of patient representatives, providing further insight into the subject matter. This is a valuable contribution as they possess a comprehensive understanding of the medical, psychological, and ethical considerations involved. Patient representatives placed particular emphasis on the advantages of avoiding a diagnostic odyssey and the significance of support systems. The results provide first insights into different views on gNBS in Germany. These views can inform the potential preparation of a gNBS program in Germany, particularly with regard to information and consent requirements. Implications for practice, such as informing and educating parents about gNBS during pregnancy, can be derived from the study.

**What is known about this topic**

Previous studies have indicated that there is significant public and—more particularly parental—interest in genomic newborn screening (gNBS). However, discrepancies exist regarding preferences for the scope of reported results, including differences between the views of healthcare professionals, parents, and the public.

**What this paper adds to the topic**

This study offers insights into various perspectives on the potential implementation of gNBS in Germany, highlighting divergent views of healthcare professionals and parents, while also introducing a novel and valuable contribution by including the viewpoints of patient representatives.

## 1 INTRODUCTION

Newborn screening (NBS) is widely regarded as one of the most effective public health programs (Botkin, 2016). NBS uptake ranges from 30% in developing countries to over 99% in industrialized countries (La Marca et al., 2023). In Europe, most countries surpass 90% uptake, with many reaching over 99%, despite non‐mandatory policies (Loeber et al., 2021). The German Society for Neonatal Screening reported a 99.98% uptake in 2020 (Brockow et al., 2022). The number of conditions screened for via biochemical analyses varies widely between countries and has changed over time with the inclusion of new conditions (Loeber et al., 2021). Currently, there is a lively discussion on the implementation of genome sequencing in NBS, i.e., genomic newborn screening (gNBS) (Baple et al., 2024; Dikow et al., 2022; Remec et al., 2021; Spiekerkoetter et al., 2023). This is because next‐generation sequencing methods have advanced rapidly, and the cost and processing time of these methods have decreased substantially in recent years, making them more applicable in healthcare settings. Genomic testing can detect a range of diseases early, enabling prompt prevention and treatment when available. However, the introduction of gNBS as a baseline screening would present clinical, legal, ethical, and psychological challenges, as detailed elsewhere (Berg et al., 2017; Dikow et al., 2022; Ditzen & Schaaf, 2023; Johnston et al., 2018; Kerruish, 2005). In their review, Downie et al. (2021) found a high level of interest (>60%) in parents and the public responding to hypothetical and actual offers of gNBS across 14 studies, although the desired level of results returned varied considerably. In contrast, in the BabySeq pilot project, less than 10% of approached parents participated in the gNBS study. The most common reasons for decline were low interest in research and study logistics (Genetti et al., 2019). Additionally, participants considered the accuracy of disease prediction by the test to be the most crucial attribute of the screening. Healthcare professionals (HP), however, tended to prefer selective disclosure of results based on clinical utility (Clark & Boardman, 2022).

Considering that NBS programs and healthcare systems are determined nationally, the implementation of gNBS in public healthcare must be evaluated on a country‐specific basis. To our knowledge, there is no research on views of relevant groups regarding the introduction of gNBS in Germany, and most research on gNBS originates from North America (Downie et al., 2021). In Germany, NBS is currently offered to all newborns, covering 19 target diseases in different pediatric fields, including metabolic diseases (Spiekerkoetter & Krude, 2022). Given the ethical complexities and societal implications that may arise from the integration of genome sequencing into current standard newborn screening programs, it is crucial to engage relevant groups in the discourse prior to any potential implementation in public health or pilot programs. Public engagement has been emphasized by initiatives such as the “Newborn Genomes Programme” and “The Generation Study” by Genomics England (https://www.genomicsengland.co.uk/initiatives/newborns/engagement), and the importance of engaging relevant groups has been highlighted by Baple et al. (2024).

Focus group discussions provide a participatory approach that facilitates and encourages involvement from different groups. Clark and Boardman (2022) reviewed previous studies of different perspectives on gNBS. In particular, the underlying original studies using focus groups focused on the perspectives of parents, clinicians, or the public. To also involve the patient perspective in this exploratory study, we conducted focus group interviews with parents (P), patient representatives (PR), and healthcare professionals (HP). Adding the PR perspective is important because this group represents those, who are actually affected by a (mis)diagnosis of a rare disease and their personal experiences could add valuable information.

## 2 METHODS

### 2.1 Participants

We conducted semi‐structured focus group interviews with parents of minors, PRs of genetic diseases (members of patient organizations), and clinically active HPs (human geneticists, pediatricians). Potential participants were approached via flyers and emails. Parents and HPs were recruited locally in Heidelberg, PRs were recruited nationwide. We used purposive sampling to select potential participants of the three groups. Representing multiple roles (e.g., physician and parent) was permitted to reflect the real setting. Being of legal age and proficiency in German were required, prior knowledge of gNBS was not necessary. A total of four focus groups were conducted online. The study was approved by the Ethics Committee of Heidelberg University Hospital (S‐7262022).

### 2.2 Instrumentation and procedures

Questions and prompts were generated based on literature research (a.o. Armstrong et al., 2022; Downie et al., 2021; Joseph et al., 2016; Lewis et al., 2016) and expert knowledge from the study team in the areas of ethics, human genetics, pediatrics, law, and psychology. Interview guides were pilot tested (three persons, one round) with no changes made afterward and are available from the authors on request. All four focus groups were conducted in German. The first two focus groups in May 2023 specifically addressed the perceived benefits and risks of a potential gNBS program in Germany, preferences regarding included diseases, reporting of findings, and data storage. The two focus groups held in June 2023 centered on the topics informed consent, decision‐making, and steps after result disclosure. We limited the study to four focus groups due to capacity constraints. We aimed to ensure sufficient depth of understanding to adequately address the study aims by incorporating diverse perspectives (HPs, PRs, parents) into the focus groups. After each focus group, the team discussed whether the questions were sufficiently explored to provide a solid basis for analysis.

Prior to the discussions, all participants signed a declaration of consent and confidentiality and provided demographic information, including age, gender, and number of children. The questions to be discussed in the focus groups were sent to participants a week in advance. Discussions were conducted online and lasted approximately 2 h each. To cater to varying levels of prior knowledge, an email was sent to participants a day before the session, which included an overview of current NBS in Germany and its potential expansion through genome sequencing. Additionally, a 15‐min introduction providing basic information on newborn screening and genomic testing was given at the start of each focus group. Participants were informed that the study focused on their perspectives on gNBS. Focus group discussions were audio‐visually recorded, and the participants were given a 100‐euro incentive. No field notes were taken. All four focus groups were moderated by the first author, ESD. She holds a Master of Science in Psychology and works as a research associate and clinical psychologist and introduced herself as such. Before the study, ESD had email contact with participants to organize the groups. ESD had pilot training, was supervised by researchers with extensive experience in focus group discussions (authors JM, SPL), and has advanced communication skills through her graduate degree in Psychology and ongoing clinical training in Psychotherapy. Additional members of the study team (1–3) silently observed and recorded the sessions without turning on their cameras.

### 2.3 Data analysis

The audio‐visual recordings were transcribed verbatim. Qualitative data was analyzed in multiple rounds of coding using a structuring qualitative content analysis that combined a deductive and inductive approach (Elo & Kyngäs, 2008; Mayring, 1994). In the first step, we familiarized ourselves with the material. Authors ESD and JM then agreed on six broad content categories. The categories were defined with the objective of reflecting the central topics of the focus group discussions and based on current debates regarding gNBS. Consequently, they are partially aligned with the interview guide, but are not identical to the questions themselves. ESD then open‐coded the transcripts within the defined content categories, and all transcripts were co‐coded by three student researchers. Disagreements were discussed in the team. All lower‐level codes were collected in an Excel spreadsheet. ESD categorized the lower‐level codes into subcategories and more detailed fine‐grained subcategories. The identified subcategories were discussed with JM and the study team to assess credibility. The team revised content categories, subcategories, and codes repeatedly during the process with constant exchange. We used QCAmap (https://www.qcamap.org/ui), an open‐access web application for systematic text analysis based on the techniques of qualitative content analysis (Mayring, 1994), and Microsoft Excel to facilitate coding, analysis, and data management. Member checking was not conducted for either the transcripts or the results. Reporting of this work adheres to the consolidated criteria for reporting formative qualitative research (COREQ) (Tong et al., 2007).

## 3 RESULTS

Of the individuals who expressed interest in participating in the study, three participants dropped out. One participant withdrew due to illness, another due to technical problems with online meeting participation, and the third participant likely forgot and did not respond to emails. The study ultimately consisted of four online focus groups with a total of 24 participants, including parents, physicians, and adult patient representatives (*n* = 8 per subgroup). Table 1 presents the characteristics of the 24 participants. We acknowledge that a strict separation of different roles was challenging as some participants held multiple roles, such as being both a P and HP. In the results section, we primarily consider each participant's primary role, which was assigned based on the participants' self‐identification or the channel through which they had been recruited.

**TABLE 1:** Characteristics of focus group participants.

Participants in total (*n*)/gender (*n*)	24/20 female, 4 male
Age in years (mean, range)	42 [28; 68]^a^
Parents (P) (*n*)	8
Number of children (mean, range)	2.3 [1; 3]^b^
Age of children in years (mean, range)	6.0 [0; 15]
Patient representatives (PR) (*n*)	8^c^
Diseases (affected or representing)	Dup15q syndrome, Li‐Fraumeni syndrome, BRCA, cystinosis, Metachromatic leukodystrophy, phenylketonuria, chronic rare diseases
Having children (*n*)	6
Health professionals (HP) (*n*)	8
Human geneticist (*n*)	3
Pediatrician (*n*)	5
Experience in years (mean, range)	10.3 [3; 31]
Having children (*n*)	4

*Note*: All participants were German.

^a^ One participant did not report her age.

^b^ Unborn children of pregnant mothers (*n* = 2) were included.

^c^ One patient representative was also a gynecologist, another had a degree in medicine and used to work in genetics research.

**FIGURE 1:**
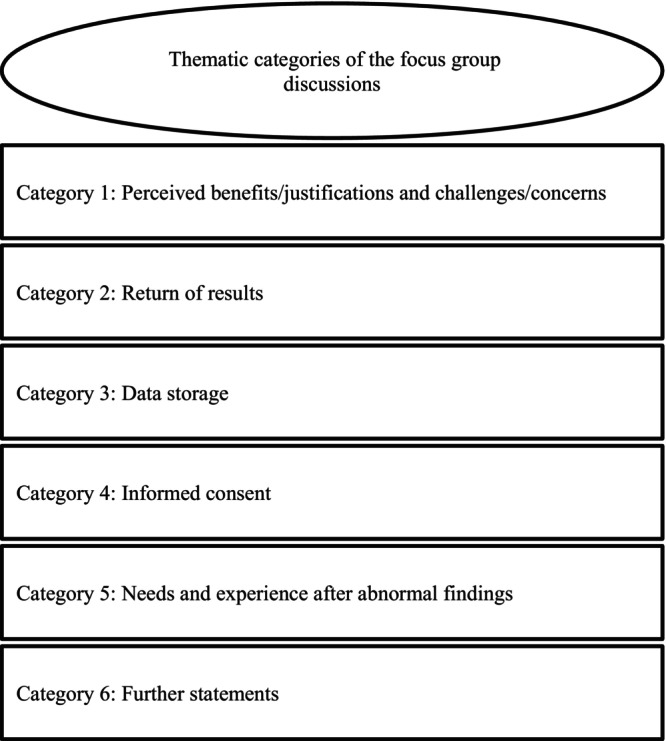
Thematic categories of the focus group discussions. Themes of each category were mentioned in all four focus group discussions.

The results of the qualitative content analysis are structured according to the six thematic categories (see Figure 1): (1) *perceived benefits/justifications and challenges/concerns*, (2) *return of results*, (3) *data storage*, (4) *informed consent*, (5) *needs and experiences after abnormal findings*, and (6) *further statements*. Within each content category, subcategories are presented, along with the total number of participants and the number of participants per role who commented on each subcategory. For illustration, quotes translated by a person with English language skills at native speaker are included. To protect privacy, only the participant's role is specified. While the discussions in Groups 1 and 2 focused on questions pertaining to content categories 1–3, and those in Groups 3 and 4 on questions related to content categories 4 and 5, all categories were addressed in each focus group discussion.

### 3.1 Content category 1: perceived benefits/justifications and challenges/concerns

Participants discussed various benefits and challenges of gNBS, including clinical and practical aspects, psychological considerations, and ethical implications. Table 2 provides an overview of the subcategories, their numerical occurrence across the three groups, and illustrative quotes. The subcategories are explained in more detail in the following.

**TABLE 2:** Data related to Content Category 1: Perceived benefits/justifications and challenges/concerns by focus group participants.

Perceived benefits/justifications	Perceived challenges/concerns
**Clinical and practical benefits** (*n* = 14: 4P, 6PR, 4HP)	**Clinical and practical challenges/concerns** (*n* = 12: 3P, 4PR, 5HP)
Early detection and actionability^a^ (*n* = 9: 2P, 5PR, 2 HP)	Patient‐related practical barriers^b^ (*n* = 6: 3P, 1PR, 2HP)
Development of treatments (*n* = 5: 2PR, 2HP, 1P)	Clinical limitations^a^ (*n* = 5: 1P, 2PR, 3HP)
Complement to NBS (*n* = 2: 1P, 1PR)	Implementation issues (*n* = 5: 2PR, 3HP)
Cost savings (*n* = 1: 1PR)	Lack of resources (*n* = 5: 3PR, 2HP)
	Alignment of a gNBS program (*n* = 5: 1PR, 4HP)
^a^Illustrative quote: “And exactly, the last thing would be therapy. There is no causal therapy, i.e. no medication that can reverse the disability, but if I can start from birth with physio, ergo, logo, where the neuroplasticity is still very high, a lot is helped.” (PR)	^a^Illustrative quote: “This is a major uncertainty that we have to take into account, so even variants that are classified as clearly pathogenic or as innocent change their classification, unfortunately you always have to look at it again.” (HP)
**Psychological benefits** (*n* = 9: 4P, 4PR, 1HP)	**Psychological burden** (*n* = 15: 7P, 6PR, 2HP)
Preparedness and coping of parents^a^ (*n* = 7: 2P, 4PR, 1HP)	Uncertainty and coping challenges^a^, ^b^ (*n* = 6: 4P, 2PR)
Reduced psychological burden (*n* = 8: 2HP, 4PR, 2P)	Emotional impact^a^, ^b^ (*n* = 6: 2P, 4PR)
Relief for affected families (*n* = 4: 1 HP, 1PR, 1P)	Postpartum period stressful^b^ (*n* = 4: 3P, 1PR)
	Burden for child (*n* = 4: 1P, 1PR, 2HP) Impact on parent–child interaction^a^, ^b^ (*n* = 3: 2P, 1HP)
	Stress for family^b^ (*n* = 1, 1PR)
^a^Illustrative quote: “But that would be an opportunity to give a disease a name at an early stage and thus prepare parents for the fact that symptoms could develop in such and such a number of years, even if there is no treatment. And of course this avoids double diagnosis and doctor hopping and such.” (PR)	^a^Illustrative quote: “If you only have one abnormal finding, it doesn't mean that the disease will break out. So, I think it's important to emphasize that you can scare parents who are experiencing a lot of stress and grief. As TB said, the parent–child relationship is different and you deal with the child differently, even though there is not even an outbreak of the disease.” (P)
**Ethical justifications** (*n* = 3, 1P, 1PR, 1HP)	**Ethical challenges** (*n* = 12, 2P, 5PR, 5HP)
Rights of child, parents' responsibility to decide (*n* = 3: 1P, 1PR, 1HP)	Rights of child^b^ (*n* = 6: 1P, 4PR, 2A)
gNBS less questionable than prenatal screening^a^ (*n* = 1: 1P)	Right of family members^a^, ^b^ (*n* = 3: 2PR, 1HP)
	Information sufficiency^b^ (*n* = 2: 1HP, 1PR)
	Risk of overdiagnosis^b^ (*n* = 1: 1HP)
	Ensuring equality^b^ (*n* = 2, 2HP)
^a^Illustrative quote: “And for me, it's not this sifting out, as when you do a alike diagnosis while the child is still in the womb.” (P)	^a^Illustrative quote: “There are exemplary cases where the family says they don't want to know the result because through that it's clear that I have it as well and I don't want to know that because I want to live my life the way I have it at the moment.” (HP)

Abbreviations: HP, health professionals; P, parents; PR, patient representatives.

^a^ Illustrative quotes are provided for selected subthemes (highlighted with ^a^).

^b^ Some challenges referred to both, worries toward gNBS as well as challenges that were expected to occur after testing.

#### 3.1.1 Clinical and practical utility versus challenges

The perceived primary clinical utility of gNBS, particularly highlighted by PRs, involved early detection and actionable information for prevention, treatment, and parental support of affected families. Furthermore, some participants anticipated that advancements in treatments would result from increased research and industry attention. From a practical standpoint, some viewed gNBS as a convenient addition to current screening practices, or as a natural progression from existing methods. Nevertheless, several clinical challenges were identified, including uncertainties in diagnosis, the identification of untreatable conditions, and the time necessary for developing new treatments. Additionally, challenges pertaining to implementation were discussed, including matters of disclosure timings and management of novel genetic variants, as well as transitioning toward expanded gNBS. During the discussion of clinical benefits and challenges, HPs and PRs representatives emphasized the importance of clarifying the role of gNBS, distinguishing it from diagnostic testing, and positioning it appropriately within newborn screening or predictive testing frameworks. Additionally, participants identified potential practical challenges for families, including financial considerations associated with gNBS, as well as potential discrimination in insurance coverage or job opportunities, costs of care, and logistical challenges in the event of abnormal findings. In a more global context, participants identified a number of systemic challenges within the German healthcare system. These include limited resources, a lack of genetic expertise among healthcare providers, and a lack of genetic literacy among the general population. Furthermore, disparities in access to medical care and centers for rare diseases across Germany were noted. Some participants expressed concerns about the time pressures associated with commercially available genetic tests.

#### 3.1.2 Psychological utility versus psychological burden

The participants identified the primary psychological benefits of the program as reduced psychological burden, preparedness, and coping for parents, along with relief for affected families through certainty and knowledge. The most frequently cited benefit of reduced psychological burden was the prevention of a diagnostic odyssey when symptoms occur. PRs shared their personal experiences with misdiagnosis, the lengthy journeys they undertook to obtain a diagnosis, and the challenges they faced in finding suitable physicians. The utility of preparedness and coping, also stated by parents, included the ability to accept the disease at an early stage, consider treatment options in a timely manner, plan for the future with certainty, and seek psychological and social support at an early stage. On the other hand, two mothers identified psychological burden as the only disadvantage of gNBS:

I've been asking myself the whole time what the disadvantages actually are. And then came to the same conclusion as the previous speaker: the psychological component.

Participants expressed concerns about potential uncertainty and coping challenges related to the onset and severity of the disease, as well as dealing with constant alertness in the event of a known high‐risk condition. They also anticipated negative emotional impact such as guilt, helplessness, fear, and anxiety in the case of abnormal findings, stress related to false positive or uncertain results, and a sense of guilt if they decided against gNBS. One participant expressed concern that educating parents about gNBS could cause unnecessary anxiety. Additionally, some participants noted that the postpartum period was already a stressful and overwhelming time, and that gNBS could add to this burden. Others were worried about the potential impact on parent–child interaction in the event of abnormal findings, such as communicating the results or perceiving the child as imperfect, and anticipated psychological burden for the child and stress for the entire family due to uncertainty and potential conflicts triggered by genetic information.

#### 3.1.3 Ethical considerations

The implementation of gNBS was considered ethically justifiable by some participants. It was argued that the rights of the newborn would not be violated because the parents, who are responsible for the child, would make decisions in the best interests of the child. Furthermore, they contended that gNBS would be less controversial than prenatal diagnostics. Nevertheless, others perceived the potential violation of the child's right to know if parents decided against gNBS or did not share the findings with their child, or if the child was not involved from a certain age. Ethical challenges at the family level included the right of other family members not to know and the potential impact of genetic information on them. Some participants identified ensuring equality as a challenge, citing the lack of resources for gNBS in certain areas. It was emphasized that it was of the utmost importance to avoid any disadvantages that may befall less privileged families and to ensure that the quality of screening is consistent regardless of location. Furthermore, there was a need to avoid overdiagnosis through gNBS and to determine what information is sufficient and necessary to know was expressed:

How much knowledge does a society need, how much knowledge does an individual need in this situation? (PR)

### 3.2 Content category 2: return of results

During the study, some participants expressed their preferences for the type of information they would like to receive, while others discussed the benefits and challenges associated with certain disclosure criteria. Discussion topics are provided in written form according to the criteria that were discussed. In general, participants expressed a preference for a combination of different categories, with actionable childhood‐onset findings being the most common (3P, 1PR).

#### 3.2.1 Age of onset

Two participants noted the importance of specifying the age of onset up to which diseases were reported in a potential gNBS (1HP, 1PR). Five participants (3P, 1PR, 1HP) considered childhood‐onset diseases to be relevant, while eight participants considered adult‐onset diseases to be less important (2PR), not necessary to know (5HP), or difficult to decide (1PR). One PR proposed limiting gNBS to diseases relevant for early childhood and conducting a rescreening in later childhood or adolescence. Reservations about adult‐onset findings were explained by legal regulations (2HP), the child's right not to know and make their own decisions (3HP), the potential burden on the child (1PR), and the challenge of how to communicate the information when the child is older (1PR).

Illustrative quote: “At a certain point, the children who are examined will have their own medical decision‐making rights. And just because the parents wanted to know at birth whether the child will have a disease at the age of 20, 30, 40 – maybe the patient wants to decide for himself at 18, I don't even want to know. I think you simply have to take this discrepancy into account, that there can be different interests.” (HP)

#### 3.2.2 Actionability

The participants expressed divergent views on the criteria of actionability. Seven participants expressed a desire to know actionable findings (3P, 2HP, 2PR), while two specified *only* actionable findings (1HP, 1P). One PR expressed a preference for both actionable and non‐actionable findings, while some remained undecided about non‐actionable findings (1HP, 1P). The preferred extent of actionability also varied. While some PRs demanded a broad scope of actionability, others limited the criterion to actionability within a specific timeframe (1P, 2HP). Reasons for preferring actionable findings included supporting the child (1PR), reducing uncertainties (1HP), and accelerating the implementation of gNBS (1HP). Arguments in favor of non‐actionable findings were understanding symptoms when they occur (1PR), life planning (1PR), and certainty (1P). A mother drew parallels between non‐actionable findings and the prenatal dilemma. Additionally, one PR prioritized actionability over certainty of prediction and age of onset:

But if I can draw consequences in some way, for example from medication to dietary changes or something, then of course I am very much in favor of it, even if there is only a certain risk, not 100% as with Chorea Huntington, but as with BRCA, for example, where I can do something, but I don't have to do it early. (PR)

#### 3.2.3 Risk of disease onset (penetrance)

HPs and parents underscored the importance of high certainty regarding disease outbreaks (2HP, 3P). They argued that families could cope better with definitive information than ambiguous data (1HP) and expressed concerns about uncertain findings that might strain the parent–child relationship due to parental anxieties (1P). One mother emphasized the importance of high certainty, especially in the context of actionable findings, whereas another mother regarded high certainty as crucial even in the absence of actionable results. Parents exhibited ambivalence regarding the threshold of certainty required for predictions, e.g., a mother stated,

I would like to know whether the [disease] has a 50% chance or more of actually occurring. 60%–70% that it will occur, then I could imagine wanting to know that. (…) I think even if it wasn't treatable. Or maybe even higher, I don't know. But I think the uncertainty is better not to know. (P)

A similar recommendation was made by a HP, indicating that the disease risk to parents was probable when the penetrance level is between 50% and 80%.

#### 3.2.4 Carrier status

Five participants shared views on the disclosure of carrier status. One mother emphasized its importance for family planning, stating:

I think this carrier status is important, for example when parents want to have further children. For example, other siblings could also be affected. (P)

However, HPs expressed concerns about disclosing carrier status, given their anticipation that almost every newborn would have a positive finding. They proposed that carrier information would be incorporated into prenatal diagnostics, rather than relying solely on NBS. Two PRs, one of whom initially supported the disclosure of carrier status, expressed concern about the potential overload of abnormal findings and considered reproduction‐focused information to be of less relevance.

#### 3.2.5 Pharmacogenetic findings

Pharmacogenetic findings provide information about the potential effects of medications, including possible adverse reactions. The perspectives on receiving these findings varied among participants and with some expressing limited familiarity with this type of information. Two participants declined to receive pharmacogenetic findings, considering them non‐targeted and more appropriate in cases of medical complications (1P, 1HP). One participant articulated this sentiment, stating:

I would want to know [pharmacogenetic findings] if I tried a medication and it didn't work and then a second one and that still didn't work – that you only then look specifically for it and not aimlessly from the beginning. (P)

Additionally, a pediatrician highlighted a specific challenge related to pharmacogenetic findings: the responsibility of physicians when complications occur in another hospital. In contrast, other participants identified significant value in pharmacogenetic findings. A mother prioritized these findings due to their potential immediate relevance in emergency situations. Furthermore, a PR advocated for the disclosure of pharmacogenetic findings at birth, citing prior medication challenges experienced by families.

#### 3.2.6 Variants of uncertain significance (VUS)

In particular, the group of HPs argued that VUS should not be reported, to which others agreed (2P, 4HP, 1PR). They were specifically concerned about uncertainties and misinterpretations among medical experts (1HP), distress among parents (3HP), and perceived no clear benefit (1PR, 1P). One HP cautioned that:

[VUS] means, above all, you really don't know what they mean and many variants will have to be re‐sorted in the future anyway. (…) you shouldn't use VUS for any kind of treatment and I can only strongly advise against communicating them, regardless of the setting, because they can lead to major misunderstandings and major misconceptions.

HPs additionally classified VUS as complex and resource‐intensive and placed them into the field of diagnostic testing and research rather than newborn screening (3HP). Nevertheless, a PR and a mother expressed a preference for being informed if the meaning of a previous VUS became known or symptoms manifested.

#### 3.2.7 Any information

A PR and a mother stated that all information was relevant to them:

For me, all things are actually important. And the families from our association or those affected agree with me that it's actually better to know everything and then decide for yourself how to deal with it. (PR)

They justified their decision by citing potential treatment options in the future, the belief that early knowledge and adaptation is beneficial, and the idea that choosing gNBS means accepting possible uncertainties and taking responsibility for handling the information. Later, the PR classified VUS and adult‐onset findings as secondary relevant. The mother subsequently advocated that physicians should be the arbiters of what information was relevant to report to parents.

#### 3.2.8 Additional considerations

In addition, participants identified further criteria for determining which information should be disclosed. For example, variants with a known family predisposition (P), using criteria from the current NBS (HP), and a prioritization of severe and rare conditions (PR). General disclosure demands included informing the child at an appropriate time (P), reporting unsuspicious findings, and establishing structures so that families are not responsible for requesting findings (HP). A holistic approach was proposed in response to the question,

What is worth knowing and what can we support well? (PR)

and it was suggested starting on a small scale in the initial stage of a potential gNBS in Germany (PR).

### 3.3 Content category 3: data storage

Table 3 presents perceived benefits, challenges, and needs of storing newborns' genome data. This includes considerations regarding data access, resources, and the use of this data for research purposes. The demand for high data protection standards and clear regulation of data access was expressed with the greatest frequency and consistency. Despite the diverse opinions on the value of storing gNBS data, practical limitations were identified, particularly by HPs. Furthermore, some parents expressed reservations about the prospect of their child's genomic data being stored in a repository. Some participants requested the ability to destroy data immediately after result disclosure, while others preferred data storage up to lifelong preservation. A mother additionally demanded to obtaining the child's subsequent consent in case of data storage.

**TABLE 3:** Data related to Content Category 3: Views of focus group participants on storage of gNBS data.

Subcategories	Illustrative quotes
Data access	
**Benefits** (*n* = 6: 2P, 3PR, 1HP) Potential future treatment Information preservation Updating information (e.g., VUS)^a^	“Just because a variant is of unclear significance now doesn't mean that it will remain so. I see great opportunities here. But for this to happen, data must be routinely reviewed and analyzed again and again.” (PR)
**Needs** (*n* = 13: 5P, 3PR, 5HP) Data protection and access^a^ Findings (not) to be stored Recall system Networking, digitalization	“The data must be protected, of course you have to check the access rights very carefully (…) that the patient has primary rights and until he/she is of legal age the parents (…) doctors should have access in case of a diagnosis, in an emergency, if an operation or medication is pending or symptoms occur.” (P)
**Challenges** (*n* = 6: 1P, 5HP) Limited future utility of stored data Establishment of recall system Security risks^a^ “Future treatment options” as storage criteria hard to implement What type of findings to store	“I do see a considerable danger if there are complete data sets of my children's genes somewhere, especially now with security gaps that are somehow alarming.” (P)
Resources	
**Benefit** (*n* = 1: 1P): Cost benefit	“If the work [genome analysis] has already been done, it would also be great for financial reasons if this remains [saved] forever.” (P)
**Need** (*n* = 1: PR): Save resources	“I think we can save a lot of resources if we really look at what [kind of databases] are already available.” (PR)
**Challenges** (*n* = 2: 1PR, 1HP): Cost, storage capacity	“What I still see as a very big problem is the amount of data we have. That is a huge capacity problem.” (HP)
Research use	
**Benefits** (*n* = 3: 1P, 1PR, 1HP) Gather more knowledge^a^ Research use general positive	“Especially in the case of rare diseases, the storage of data in anonymized form can help to know how frequently this disease actually occurs. So, what is the incidence, is it always penetrating (…)” (PR)
**Needs** (*n* = 6:1P, 1PR, 2HP) Anonymized/pseudonymized Government regulation Mutual exchange^a^ Clarify data storage for patient care vs. research use	“Regarding data donation (…) we are setting the minimum requirement that those who donate their data, especially for research, also receive feedback on what happens to this data. Is there a research project, what is the result (…). I think that also promotes compliance, the willingness on the part of the patients to be interested in research and to provide data if there is a mutual exchange.” (PR)

Abbreviations: HP, health professional; P, parent; PR, patient representative.

^a^ Illustrative quotes are provided for selected subthemes (highlighted with ^a^).

### 3.4 Content category 4: informed consent

Participants identified essential information for decision‐making about gNBS and discussed potential impacts on their hypothetical decisions. They also shared their experiences with education and consent within the current NBS system and discussed structural needs for improvement. Subsequently, the topics discussed are provided in written form.

#### 3.4.1 Informational needs

Participants expressed a need for basic information about the procedure, including what is being screened, basic genetics knowledge, a summary of included diseases or conditions, and the likelihood of actionability (2P, 1PR, 2HP). They also wanted an evaluation of gNBS (e.g., benefits and limitations) and information on procedures and potential consequences post‐testing, such as the impact on families, consequences of declining testing, communication of results, and the availability of contact points and psychological counseling in case of abnormal findings.

#### 3.4.2 (Psychosocial) impact on decision‐making

Factors anticipated to influence parents' decision‐making regarding gNBS were primarily social and environmental (3PR, 3HP, 6P), including family and friends' behavior or advice, trends, and social media. Additionally, experience with genetic diseases in the family, origin, religion, and culture were assumed to play a role. Two mothers underscored the significance of physicians' counsel and opinion and their rapport with the medical practitioner. Conversely, one HP believed the decision should be made exclusively by the parent couple. One focus group postulated a female dominance in decision‐making about gNBS. Eight participants identified specific attitudes and values that would be pertinent to their decision‐making process. These included “better to know than not to know” or “being the type of person who wants to know things,” willingness to cope with difficulties, and parental responsibility for the child. Conversely, it was posited that a desire to avoid potential negative outcomes or fear of specific findings and loss of control, as well as a tendency to take things as they come, could influence the decision against gNBS. In general, it was observed that attitudes toward genomics and technological advances, as well as behavior in other health decisions, could influence the decision to undergo or decline gNBS. The two mothers engaged in a hypothetical reflection on their decision‐making strategies, including their own internet research, partner discussions, and intuitive or rapid decision‐making processes and they anticipated a more extensive search for information with their first child. A PR emphasized the importance of considering the potential impact on relatives when making decisions about gNBS.

#### 3.4.3 Consultation and consent framework

The topic of advice on and consent to a gNBS was deemed to be of significant importance to the participants, who discussed a number of structural and communication needs. Thirteen participants indicated a need for early, optimal prenatal education to allow parents sufficient time to consider gNBS (6P, 3HP, 4PR). In regard to the question of who should provide education, a variety of options were discussed, including gynecologists and midwives as an appropriate source for information during prenatal care, human geneticists, pediatricians, or the primary care physician. Two HPs emphasized that the responsibility for providing education should not be solely placed on human geneticists due to capacity limitations. A PR highlighted the importance of informants having a nuanced understanding of gNBS. Two mothers discussed the significance of individual differences in medical affiliation, such as only being in contact with midwives instead of gynecologists, which should be taken into consideration. Participants identified a need for standardized official information, educational videos, informative online portals, accessibility and simplified language, in‐person consultations instead of information sheets, the chance to have a second consultation if needed, and—as a large‐scale strategy—to increase genetic literacy among resident physicians and the general population, e.g., by integrating education about gNBS into schools (2P, 4PR, 2HP).

The participants expressed a desire for active consent and emphasized the importance of free choice (2HP, 4P, 2PR). Additionally, flexibility was perceived as valuable, including the option to request insight into findings initially opted out for at a later time, the ability to change consent, and the option to be contacted if non‐actionable conditions become actionable. There was support for offering several individual options, including the ability to opt out of storing their child's data, to exclude low penetrant findings, and to make individual decisions regarding non‐actionable findings. However, one PR expressed concern about the necessity for professional consultation and time when considering individual options, and it was emphasized that reducing complexity (HP) was essential. Moreover, the necessity for communication during the education about gNBS included the provision of information without the intention of influencing the couple's decision, the encouragement of the couple to make the decision independently, the allocation of sufficient time for physicians to educate, and the addressing of parents' concerns during the consultation session (2PR, 1P, 2HP).

#### 3.4.4 Experience with current NBS

Seven participants shared their experience with the current NBS program in Germany. Some parents were uncertain as to whether NBS had been conducted or if they had been educated about it, or were unaware of the specific screening conditions their child had undergone. One mother recalled feeling overwhelmed by the multitude of medical decisions that needed to be made during pregnancy and feeling incapable of making any decisions herself. She perceived that her consent for NBS had been assumed by the medical personnel. A PR and father expressed appreciation for having been informed in advance. He recognized that the circumstances surrounding the birth of a child are exceptional and that parents may not critically question their consent. Pediatricians acknowledged that parents often lack sufficient understanding when informed about NBS after birth. However, they have never encountered parents who have rejected NBS.

### 3.5 Content category 5: needs and experience after abnormal findings

Participants expressed structural, informational, and supportive care needs when gNBS would reveal abnormalities in their newborns. Additionally, some shared their own positive and negative experiences with situations related to abnormal genetic findings. Table 4 provides a more detailed account of the subcategories, including their frequencies and illustrative quotes.

**TABLE 4:** Data related to Content Category 5: needs and experience after abnormal finding.

Needs
**Structural needs** (*n* = 13: 4P, 6PR, 3 HP)
Specialists, multidisciplinary team
Local contact^a^
Healthcare architecture ideas: networks, genetic counseling^a^
^a^Illustrative quote: “I'm imagining it right now, having two children at home, just having a third and having to travel across the country to speak to any expert. Perhaps it would be good to have the networks primarily via telemedicine, both between doctor and patient and between local doctor and rare disease expert. We should try to do as much as possible at a local level to make life easier for first‐time parents.” (HP)
**Informational needs** (*n* = 5: 1P, 3PR, 1HP)
Detailed explanations of disease/findings, updates
Counseling about therapeutic management, services
Relevance for further children
Illustrative quote: “[That you] can really get to someone who knows about this disease and can say in the third sentence: This is bad now but I could suggest that you can do this and this. From the point of view of parents and self‐help organizations, this is what helps.” (PR)
**Supportive care needs** (*n* = 14: 4P, 5PR, 5HP)
Long‐term support of family, holistic care^a^
Trusted physician
Patient‐centered communication
Empathetic medical contact person
Communication to children
^a^Illustrative quote: “I think it's just incredibly important to have a contact person you can rely on, not just for the moment, but for the whole time you want to be well looked after. That wouldn't just be the time when I get the diagnosis, but basically also throughout my child's childhood and adolescence.” (PR)

Experience after abnormal finding (*n* = 7: 1P, 5PR, 1HP)
Self‐help organizations helpful
Negative impact on social relations and family challenging^a^
Negative experience with communication at counseling
Lack of psychological support
^a^Illustrative quote: “A woman also said to me, I've been your best friend until now, but now you have a sick child, I can't stand that, I don't want to see you anymore.” (PR)

Abbreviations: HP, health professional; P, parent; PR, patient representative.

^a^ Illustrative quotes are provided for selected subthemes (highlighted with ^a^).

### 3.6 Content category 6: further statements

A total of 13 participants expressed support for gNBS (4P, 4PR, 6HP). However, specific conditions were requested, such as offering gNBS as part of the current NBS program (P), implementing a lifelong screening program that includes not only gNBS but also other types of screening (PR), expanding the availability of genetic diagnostics instead of mandating numerous conditions in NBS (HP), providing gNBS free of charge, and avoiding obligations toward insurances (P, PR). Participants emphasized that there was a need for greater clarity regarding the definition of gNBS, particularly in terms of what gNBS would cover. One geneticist stated,

I would be against a newborn screening that leaves us with more questions than solutions. (HP)

Three participants expressed less support or opposition to gNBS: One mother initially was ambivalent and finally declined gNBS for her own children. She favored current NBS, but supported gNBS as optional offer. Another mother initially opposed gNBS and expressed a preference for diagnostic testing when symptoms occur, but later agreed that gNBS could be helpful under conditions similar to current NBS. A PR opposed gNBS as a standard program for all newborns and supported testing when a treatable condition was suspected. Overall, maintaining a holistic perspective on gNBS was stressed by participants (HPs and PRs), underscoring the significance of addressing broader health impacts. Participants emphasized the importance and complexity of the gNBS topic in healthcare policy and practice.

## 4 DISCUSSION

Given the current debates surrounding the introduction of genome sequencing in newborn screening, our objective was to gather diverse perspectives in Germany prior to its potential implementation. Our findings indicate that the participants primarily expect gNBS to improve early detection and timely treatment, accelerate the development of treatments for rare diseases, and prepare parents better about their child's potential disease risks. Nevertheless, they anticipated numerous challenges, especially concerning the practical design, implementation, and establishment of education and support structures, alongside concerns about data protection and psychological and ethical implications for families. Ambiguities, both among and within individuals, mainly revolved around the understanding or preferred scope of “actionability,” the prioritization of actionability versus penetrance and age of onset, and the timing of disclosure of findings. Participants preferred receiving prenatal education on gNBS, which differs from current NBS practices. Additionally, it was highlighted that the offer should be optional, and comprehensive support and guidance from specialists and a multidisciplinary team, including psychological support, should be guaranteed in case of abnormal findings.

A prominent theme throughout the topics was a tension between the need or hope for certainty amidst multiple uncertainties that participants were concerned about, similar to those in previous North American studies (Downie et al., 2021; Ulph & Bennett, 2023). It is of significant importance to examine this aspect, as uncertainty and ambivalence might cause reservation and discomfort. In fact, two distinct types of uncertainty can be distinguished. The first pertains to the prospective implementation of gNBS as a future scenario, necessitating clear guidelines and protocols to shape its potential introduction. The second type of uncertainty stems from individual and intraindividual ambivalence, influenced by psychological factors and concerns. This aspect underscores the need for comprehensive research and education initiatives to address psychological implications.

### 4.1 gNBS as future scenario

The unclear imagination of gNBS was a difficulty for some participants in the discussion and most participants emphasized the need for clearly and comprehensively defined conditions of a gNBS program, which is still under development. Participants' discussions about result disclosure considered legal requirements to only screen children for childhood‐onset diseases and ethical considerations such as equal access to follow‐up care in case of positive findings, children's rights against testing for adult‐onset diseases, the desire for clear outcomes, balancing child protection with respect for autonomy, and the potential parental benefits, including family planning information. These factors are already discussed in empirical and ethical literature on gNBS (Downie et al., 2021; Tarini & Goldenberg, 2012) and need to be evaluated when potentially introducing a gNBS program in Germany. Nevertheless, it can be expected that even after a potential implementation, the process and program of gNBS will continue to evolve, just as the current NBS program has continuously changed in the past.

Furthermore, specifics of data storage in the context of gNBS remain uncertain and require further examination and clarification. Mixed perspectives on data storage were evident in our focus groups, aligning with findings from previous studies in this field that indicate hopes regarding the utility of stored data, alongside concerns about data misuse, appropriate storage modalities, and data volume (Cao et al., 2023; Clark & Boardman, 2022; Joseph et al., 2016). Some of the topics discussed in the context of data storage can be considered as “future scenarios” or “wishful thinking,” as one HP expressed. Examples include needs for internationally networked databases for gNBS data, electronic patient records, continuous reclassification of VUS, including a recall system. It was highlighted that data access must be regulated, with a particular focus on data protection when implementing data storage within gNBS.

### 4.2 Intra‐ and interindividual uncertainty and ambivalence

Although there was a hope for certainty through gNBS, particularly among the parents, there were concerns about the potential psychological impacts of gNBS. These concerns included anticipated uncertainty with abnormal findings, especially during the stressful postpartum period. Other concerns were the impact on the family, coping with information, communicating with the child, the relevance of the test for future children, and strain on interpersonal relationships. Contrary to these expectations, a large North American study found no significant psychological distress associated with gNBS implementation (Pereira et al., 2021). It remains unclear whether concerns about uncertainty would negatively affect parents if gNBS were implemented in Germany. However, addressing potential psychological concerns during education and follow‐up processes is essential.

Ambivalence regarding result disclosure was another significant topic, particularly concerning the actionability of findings and the prevalence of conditions. Discussions included varying perspectives on whether non‐actionable findings should be reported, potentially influenced by different perceptions of the informational burden and interpretations of “actionability.” This finding is consistent with previous research: Tarini et al. (2009) found that approximately one‐third of parents were interested in untreatable diseases from predictive genetic testing, another third were undecided, and another third were opposed to it; additionally, Downie et al. (2020) reported that of the 68% of parents who opted for additional findings in newborns with congenital deafness, 41% chose non‐actionable findings.

Determining appropriate prevalence levels as thresholds for reporting findings has also been challenging, with considerable ambiguity about what level of risk is considered sufficiently high to warrant reporting. Notably, some participants suggested a lower threshold of 50%, reflecting maximum uncertainty regarding disease probability. Others expressed a preference to receive all possible information, thus including uncertain findings. In summary, intraindividual ambivalence suggests that if a gNBS program is implemented in Germany, it will be essential to provide parents with comprehensive education about gNBS and support them in identifying their values.

### 4.3 Views of different groups of participants

#### 4.3.1 Healthcare professionals (HPs)

In the course of our focus group discussions, the group of HPs especially highlighted a number of challenges and limitations associated with the implementation process. These included issues related to data evaluation and interpretation, healthcare system capacities, and data storage. In addition, based on their experiences with patients, they emphasized the importance of clear consequences of reported findings and voted to minimize uncertainty in the decision to disclose results in order to reduce anxiety and facilitate result handling. In general, they favored adhering to the existing criteria of NBS in Germany. This is consistent with the review by Clark and Boardman (2022), which depicts that HPs prioritize disclosure of specific results based on clinical need and are more aware of the limitations of gNBS than parents or the public.

#### 4.3.2 Parents

What particularly characterized the group of parents was their hope of gaining certainty through gNBS and being able to mentally prepare early for potential illnesses, a sentiment also reported in North American studies (Pereira et al., 2021). Previous literature identified that parents and the public were more “enthusiastic” about gNBS than medical personnel, holding a broad view of future and current benefits for the family and wider society, and empowerment through non‐actionable findings alongside actionable early‐onset findings (Clark & Boardman, 2022). On the one hand, parents in our focus groups initially were less aware of challenges and uncertainties than HPs. However, they expressed particular concern about their ability to cope with potential findings. During the course of the discussions, some parents exhibited a degree of uncertainty and caution, particularly with regard to the psychological implications of the procedure, rather than displaying a complete enthusiasm for it. This could be partially attributed to the heterogeneous composition of the groups, specifically the fact that HPs emphasized challenges and advocated for caution. Additionally, parents emphasized the potential influence of social norms and doctors' recommendations on their decision‐making regarding gNBS. The impact of perceived normative influence was also reported in previous research on parents' intention to enroll children in an expanded newborn screening program (Paquin et al., 2016).

#### 4.3.3 Patient representatives (PRs)

The group of PRs exhibited a high level of knowledge on the subject of rare diseases. PRs especially emphasized the advantages of early detection and diagnosis, drawing upon their own experiences, which frequently entailed a diagnostic odyssey. This experience was also reported by parents of children with newborn screening‐related conditions (Bush et al., 2022). They expected reduced psychological burden through early knowledge. On the other hand, they were aware of challenges such as the emotional impact of the information, effects on social relationships, and the rights of the child and other family members (not) to know. They uniformly stressed the critical need for support following abnormal findings, as also highlighted by Bush et al. (2022). This included local contacts, rapid referral to specialists, early information about the diagnosis and options, empathic contact with doctors, time and long‐term support for affected families, and connecting families with self‐help groups to facilitate peer support. However, the group of PRs was also heterogeneous: While some were strongly in favor of gNBS and in particular for the broadest possible results feedback and broad definition of actionability, one PR was also against gNBS for all newborns and instead favored faster genomic testing when a genetic condition is suspected. PRs were less represented in previous studies and were not specifically examined in Clark and Boardman's review (2022). Our study benefited greatly from the input of this group, since they contributed valuable insights into their own experiences with rare diseases and offered a holistic perspective that combined expert information about these conditions with the needs of patients and families.

### 4.4 Study limitations

Focus group studies, including ours, typically have small sample sizes and do not aim to provide a representative demographic cross‐section. Instead, their goal is to gather detailed insights into participants' views and thoughts on specific subjects. Therefore, the results are not representative of all HPs, PRs, and parents in Germany. A further limitation of the study is that we did not explicitly address data saturation through structured methods, such as code frequency counts across focus groups. However, the concept of saturation has been criticized because it implies that no new insights can be gained after a certain point in time, which may not always be feasible in qualitative research (Saunders et al., 2018; Wainstein et al., 2023). Instead, we focused on achieving sufficient depth of understanding to meet the study aims by incorporating diverse perspectives (HPs, PRs, parents), to ensure a wide range of experiences and viewpoints were captured. Moreover, the sample was characterized by a high level of education and consisted of 80% females, consistent with previous findings on gNBS engagement (Downie et al., 2021), suggesting a potential gender bias in medical decision‐making for children. It is important to note that HPs and parents were not recruited on a national scale, which may limit the generalizability of the findings. We did not find any studies addressing regional differences in attitudes toward genetic testing in Germany. A study from Turkey suggests that urban residents exhibit more favorable attitudes and possess greater knowledge about genetic testing compared to their rural counterparts (Altan & Çam, 2020). However, rurality did not significantly impact attitudes toward genetic tumor testing after adjusting for education and income (DiBiase et al., 2023), and enrollment rates in a large hospital system's whole genome sequencing program were actually higher than average for rural areas (Dickey et al., 2023). Regional differences in Germany have been observed regarding patients' preference for participating in decision‐making, with less interest among residents of the eastern compared to the western parts of the country (Hamann et al., 2012); however, this trend was not evident in diabetes patients (Drewelow et al., 2018). It remains unclear whether such differences may exist regarding decision preferences for or against gNBS. In sum, the data on regional differences is complex, and more research is needed regarding gNBS. Educational disparities may play a more significant role than regional differences in evaluation of gNBS, as studies indicate that individuals with higher levels of education tend to be more open to genetic testing and gNBS specifically (Balck et al., 2009; Prosenc et al., 2023). Another aspect that makes it difficult to interpret the results is that some participants had multiple roles (e.g., as parents and as HPs). This could lead to their perspectives not being clearly assigned to one participant group or the other, adding another level of complexity to their views.

### 4.5 Practice and research implications

The findings of this study highlight several critical considerations for the structuring of a gNBS program in Germany. It is essential to adopt a holistic approach, commencing with comprehensive education. This should include prenatal education for parents, in addition to improved genetic literacy among HPs and the public. General practitioners, gynecologists, and midwives, among others, have been identified as key sources of information. It is therefore crucial that they are adequately trained to guide parental decisions effectively, as previously suggested (Clark & Boardman, 2022). It is similarly important to consider public education, as social trends and peer opinions can influence parental decision‐making regarding gNBS (see also Paquin et al., 2016). Educational materials should be transparent and balanced, as previous research shows that transparent information about NBS and dried blood spot storage during prenatal care increases acceptance (Rothwell et al., 2016), and presenting both benefits and drawbacks is recommended to build trust (Blastland et al., 2020). It is further crucial to address parents' concerns as well as individual needs. The perception of “sufficient information” may vary among individuals. Adaptive decision aids, as previously suggested, could be beneficial in this regard (Downie et al., 2021; Stark & Scott, 2023).

Regarding consent procedures following education, participants expressed needs of flexible consent in gNBS to accommodate evolving preferences and reconsiderations regarding previously declined findings. While flexible consent was supported in previous studies too, concerns about its complexity persist (Downie et al., 2021). Care should be taken to ensure that parents are not overwhelmed by the consent process. The desire to opt out of storing gNBS data about their child was frequently expressed by participants, reflecting findings from UK‐based studies on NBS consent and highlighting the importance of parental autonomy in data storage decisions (Ulph et al., 2020).

Following up on gNBS results is critical, ensuring that abnormal findings are managed with appropriate medical and psychological support. Although Germany has a commendable healthcare system, a truly holistic approach also requires ensuring equity throughout the process, as emphasized by previous literature as well (Friedman et al., 2017). This includes equal access to information materials, consistent quality in data interpretation, and free access to gNBS and potential follow‐up care. Participants' emphasis on equality of access to follow‐up care and screening in the context of a social insurance‐based healthcare system in Germany is noteworthy and indicates the importance of informing parents that the program costs are covered by the healthcare system.

This study provides foundational insights into concerns and needs regarding a potential gNBS program in Germany. Future research should include quantitative surveys to explore public attitudes and more nuanced parental preferences toward gNBS in Germany. Additionally, analyzing parents' emotional states in relation to gNBS is crucial, as emotional factors can significantly impact medical decision‐making (Etchegary, 2004; Power et al., 2011). Further investigation into topics such as required penetrance and actionability, which were associated with intra‐ and interindividual ambivalence, is warranted.

## 5 CONCLUSIONS

This study presents various perspectives on a potential gNBS program in Germany. It is part of the research project “NEW_LIVES: Genomic NEWborn Screening Programs – Legal Implications, Value, Ethics and Society,” which aims to develop specific recommendations for a potential gNBS program in Germany. The study highlights a variety of relevant topics related to gNBS, including education, implementation, data storage, and follow‐up care, from the perspectives of healthcare professionals, patient representatives, and parents. It covers clinical, practical, psychosocial, and ethical aspects in a holistic manner and reflects key issues from the international debate. The study emphasizes the tension between the hope and need for certainty facing many uncertainties currently associated with gNBS. Identifying uncertainties and addressing them in implementation and education is crucial. Overall, the focus group discussions revealed a general impression of gNBS that can be described as cautiously positive.

## AUTHOR CONTRIBUTIONS

Conceptualization: BD, CPS, EW, JM, KA, SK; Data curation: ESD; Formal analysis: ESD, JM; Funding acquisition: BD, CPS, EW, KA, JM, SK; Investigation: ESD; Methodology: ESD, JM, KA, SPL; Project administration: BD, EW, JM; Resources: BD, EW; Software: not applicable; Supervision: BD, EW; Validation: ESD; Visualization: ESD, JM; Writing—original draft: ESD; Writing—reviewing & editing: BD, CPS, ESD, EW, JM, KA, SK, SPL. ESD confirms that she had full access to all the data in the study and take responsibility for the integrity of the data and the accuracy of the data analysis. All the authors gave final approval of this version to be published and agree to be accountable for all aspects of the work in ensuring that questions related to the accuracy or integrity of any part of the work are appropriately investigated and resolved.

## CONFLICT OF INTEREST STATEMENT

All authors declare that they have no conflict of interest.

## ETHICS STATEMENT

This study was approved by the Ethics Committee of Heidelberg University Hospital (S‐726 2022).

Human Studies and Informed Consent: Approval to conduct this human subject research was obtained by the ethics committee of the medical faculty of the University of Heidelberg. All procedures followed were in accordance with the ethical standards of the responsible committee on human experimentation (institutional and national) and with the Helsinki Declaration of 1975, as revised in 2000. Informed consent was obtained from all patients for being included in the study.

Animal Studies: No non‐human studies were carried out by the authors for this article.

## Data Availability

Data that support the findings of this study are available on request from the corresponding author. The data are not publicly available due to privacy restrictions.
